# A novel β2-AR/YB-1/β-catenin axis mediates chronic stress-associated metastasis in hepatocellular carcinoma

**DOI:** 10.1038/s41389-020-00268-w

**Published:** 2020-09-24

**Authors:** Jinxia Liu, Lishuai Qu, Chunhua Wan, Mingbing Xiao, Wenkai Ni, Feng Jiang, Yihui Fan, Cuihua Lu, Runzhou Ni

**Affiliations:** 1 Department of Gastroenterology, Affiliated Hospital of Nantong University, Nantong, China; 2 Department of Nutrition and Food Hygiene, School of Public Health, Nantong University, Nantong, China; 3 Department of Immunology, School of Medicine, Nantong University, Nantong, China

**Keywords:** Cell growth, Cancer microenvironment

## Abstract

β-Adrenergic receptor (β-AR) signalling is strongly associated with tumour progression by the coupling of β-ARs with either a G protein or β-arrestin; however, the related mechanism underlying hepatocellular carcinoma (HCC) metastasis is not clear. Here, we reveal that the transcription factor Y-box binding protein 1 (YB-1) interacts with β2-adrenergic receptor (β2-AR) following stimulation with the agonist isoproterenol (ISO). Clinicopathological analysis demonstrated that β2-AR is significantly correlated with YB-1, which favours the progression of HCC. The binding of YB-1 with β2-AR resulted in YB-1 phosphorylation at serine 102 (S102) via the β-arrestin-1-dependent activation of the PI3K/AKT pathway, followed by the translocation of YB-1 to the nucleus to carry out its tumour-related function. β2-AR-mediated activation of YB-1 facilitated epithelial-to-mesenchymal transition (EMT) and HCC metastasis. The interference of YB-1 expression significantly attenuated liver tumour metastasis induced by chronic stress. Analysis of the transcriptional profile and chromatin immunoprecipitation (ChIP) identified β-catenin as a crucial target of YB-1. Our results unveiled a novel β2-AR-mediated regulatory axis in HCC metastasis that might be helpful for the development of HCC therapeutics.

## Introduction

Liver cancer, which has an incidence of ~850,000 new cases every year, is the second leading cause of cancer-related death worldwide^1^. Hepatocellular carcinoma (HCC) is the most common primary liver cancer^2^. Despite an increasing number of treatment options for HCC, the overall prognosis of this disease remains unsatisfactory due to tumour metastasis^3^. HCC metastasis is attributed to two models: a genetic model based on genomic mutations and an epigenetic model defined mainly as modifications in gene expression that do not change the DNA nucleotide sequence^1^. Both the genetic and epigenetic models of HCC metastasis complement each other, as genetic alterations result in the formation of a metastatic cell phenotype early in tumorigenesis, while epigenetic modifications induced by external signals such as chemokines and neurotransmitters facilitate the manifestation of such a phenotype^4^. A broader interpretation of this epigenetic model of metastasis results from the regulation of the migratory activity of tumour cells by G protein-coupled receptors (GPCRs). Once GPCRs bind with potential ligands, they directly or indirectly activate intracellular protein kinases, subsequently triggering a complex series of invasion/metastasis-related cascades^5^. Stress-induced neurotransmitters released from the neuroendocrine system are prominent GPCRs ligands. They not only induce the dissemination of metastatic cells from the primary tumour but also guide these cells in a selective and organ-specific way^4^. These neurotransmitters have been found to participate in the metastasis of multiple types of tumours, including breast, colon and small-cell lung carcinoma (SCLC), suggesting that psychosocial factors favour the progression of cancer^6–11^. However, the crosstalk between these factors and the initiation and progression of HCC metastasis remain unclear.

The catecholamines epinephrine and norepinephrine, which are classic stress hormones, bind with β-adrenergic receptors (β-ARs), members of a family of seven transmembrane GPCRs. β-ARs, especially β2-adrenergic receptor (β2-AR), are aberrantly expressed in a variety of tumour tissues, where they mediate malignant cancer cell behaviours^12,13^. Abundant β2-AR expression is closely correlated with poor clinicopathological features, tumour recurrence, metastasis, and reduced survival^14–17^. Therefore, β2-AR is a cancer-relevant biomarker in carcinogenic processes. Due to the clinical significance of β2-AR expression in tumour patients, different antagonists of β2-AR, such as propranolol, atenolol, and ICI118,551, began to be gradually tried for cancer treatment^18^. However, the inhibitory effects of these β-AR blockers vary dramatically^18^. Elucidation of the β2-AR functional domain and the mechanism of its signal transduction would be beneficial for the development of pharmaceutical tumour therapies.

As a transmembrane receptor, β2-AR initiates intracellular signalling cascades, including the cAMP/PKA, MAPK/ERK1/2, p38/MAPK, PI3K/AKT, VEGF and Src/STAT pathways, through an allosteric mechanism^19^. Additionally, this receptor can induce epidermal growth factor receptor (EGFR) dimerization, tyrosine autophosphorylation and EGFR internalisation, a mechanism of ERK1/2 transactivation that stimulates its phosphorylation^20^. Several signal transducing adaptor proteins, such as β-arrestin, are recruited to activate assorted intracellular proteins that carry out a broad range of functions^21^. In the context of psychological stress, the β2-AR-dependent interaction between β-arrestin-1 and p53 leads to the Mdm2-mediated degradation of p53 and the accumulation of DNA damage^22^. Chronic stress also promotes HIF-1α expression through a β2-AR-dependent pathway to drive cancer progression. However, the regulatory mechanisms by which β2-AR promotes HCC progression have not been fully clarified.

To search for the crucial factor(s) involved in β2-AR-regulated HCC metastasis, we performed immunoprecipitation-mass spectrometry (IP-MS) analysis of HCC tissues. A vital oncoprotein, Y-box binding protein 1 (YB-1), was shown to interact with β2-AR in HCC cells. The pro-metastatic effect of YB-1 on HCC cells was regulated by β2-AR via the phosphorylation of serine 102 (S102) in YB-1, which facilitates YB-1 nuclear localization and activity^23,24^. In the present study, stimulation of β2-AR with isoproterenol (ISO) induced the phosphorylation of YB-1 at S102 and the subsequent β-arrestin-dependent nuclear translocation of YB-1 into HCC cells. YB-1 drove the progression of HCC cells through the transcriptional activation of β-catenin. These findings delineate a novel mechanistic link between chronic psychological stress and tumour progression and open up new avenues for the development of therapies to treat tumour metastasis.

## Materials and methods

### Patients and clinical samples

A total of 300 adult patients with HCC who underwent curative hepatectomy at the Affiliated Cancer Hospital of Nantong University (Cohort I, *n* = 200) and the Affiliated Hospital of Nantong University (Cohort II, *n* = 100) between January 2004 and December 2009 were included in this study. The study was approved by the Research Ethics Committee of the two institutes in accordance with the Helsinki Declaration, and written informed consent was obtained from every patient. Pre-operative clinical diagnoses of HCC met the diagnostic criteria of the American Association for the Study of Liver Diseases. These inclusion criteria were (a) a distinctive pathologic diagnosis, (b) no pre-operative anticancer treatment or distant metastases, (c) curative liver resection, and (d) the availability of complete clinical-pathologic and follow-up data. The pathological tumour-node-metastasis (pTNM) classification of HCC was based on the staging system from The American Joint Committee on Cancer/International Union against Cancer (6th edition, 2002). Differentiation statuses were graded according to the method of Edmondson and Steine. The main clinical and pathological characteristics of both cohorts are shown in Supplementary Table S1.

### Tissue microarray (TMA) and immunohistochemistry

Immunohistochemistry (IHC) staining intensities were graded from 0 to 2 (0, negative; 1, weak; 2, strong). The staining extent was graded from 0 to 4 based on the percentage of immunoreactive tumour cells (0, 0%; 1, 1–25%; 2, 26–50%; 3, 51–75%; 4, 76–100%). The final scores for the specimens calculated from the staining intensity score and the staining extent score ranged from 0 to 8. Based on the final scores, the IHC results were classified as indicating either low expression (<4) or high expression (≥4). The above procedure of evaluation was performed by three independent pathologists with a multihead microscope blindly, and a consensus was achieved among the three pathologists.

### Cell culture and cell stimulation

Human HCC cell lines (LM3, SMMC-7721, HepG2, SK-Hep1, QSG-7701, PLC, MHCC-97H), a normal liver cell line (LO2), and HEK293 cells were obtained from the Institute of Cell Biology and cultured in Dulbecco’s modified Eagle’s medium (DMEM; Sigma Chemical) supplemented with 10% foetal bovine serum (FBS; HyClone), 100 U/ml penicillin, and 100 μg/ml streptomycin in an incubator with 5% CO_2_ at 37 °C. The source of cell lines were identified and they were recently authenticated by STR profiling and tested for mycoplasma contamination. A 10 μM ISO solution was prepared fresh for every experiment by dissolving ISO bitartrate salt (Sigma) immediately before stimulation. ICI118,551 (ICI, 10 μM), 5-(2-benzothiazolyl)-3-ethyl-2-(2-(methylphenylamino)ethenyl)-1-phenyl-1H-benzimidazolium iodide (AKTi, 1 μM) or LY294002 (10 μM, Sigma) were added to the culture medium for 30 min before stimulation with ISO.

### Orthotopic xenograft tumour model and bioluminescent imaging

Forty 12-week-old athymic female BALB/C nude mice were housed under standard conditions and cared for according to institutional guidelines for animal care. All animal experiments were approved by the Committee on the Use of Live Animals in Teaching and Research, Nantong University. The mice were randomly divided into four groups. A total of 1 × 10^6^ cells in 50 μL of PBS were injected into the left hepatic lobes of the nude mice. The restraint-stress procedure refers to a physical restraint system for 2 h each day described in previous studies^10,16^. To track tumours in vivo, the transplanted cells were infected with firefly luciferase-expressing lentiviral particles (Research Science). Tumour formation and metastases were imaged by bioluminescence using an IVIS 100 Imaging System (Xenogen). Luminescent images were captured using the desired exposure times (1–60 s). The resulting greyscale photographic and pseudo-coloured luminescent images were automatically superimposed using IVIS Living Image (Xenogen) software. Four weeks following cell transplantation, the mice were sacrificed, and their organs were dissected and subjected to standard histological examination. The procedure were performed blindly.

### Immunoprecipitation mass spectrometry (IP-MS) analysis, western blotting, immunoprecipitation, immunofluorescence assays, GST pull-down assays and quantitative real-time PCR

The experimental procedures were performed as previously described^25^. The antibodies used are listed in Supplementary Table S2. The primers used for quantitative real-time PCR (qRT-PCR) are listed in Supplementary Table S3. Microarray analysis was performed by GMINIX Co. (Shanghai, China)^26^.

### ChIP assay

Chromatin immunoprecipitation (ChIP) assays were performed using a Pierce Agarose ChIP kit (Thermo Fisher Scientific, Rockford, IL) in accordance with the manufacturer’s protocol. The primers used for the ChIP assay are listed in Supplementary Table S3.

### Statistical analysis

Quantitative variables were compared using Student’s *t*-test, and qualitative variables were compared using the Pearson *χ*2 test or Fisher’s exact test. Kaplan–Meier analysis was used for survival analysis, and the log-rank test was used to compare differences. The Cox proportional hazards model was used to determine independent factors that influenced survival and recurrence based on variables identified as significant by univariate analysis. All analyses were performed using SPSS^®^ software (version 13.0, SPSS Inc., Chicago, IL, USA). *P* values < 0.05 indicated statistical significance in all analyses. All the experiments were carried out on at least three separate occasions.

## Results

### Identification of a β2-AR-interacting oncoprotein and clinicopathological correlation analysis in HCC

**Fig. 1:**
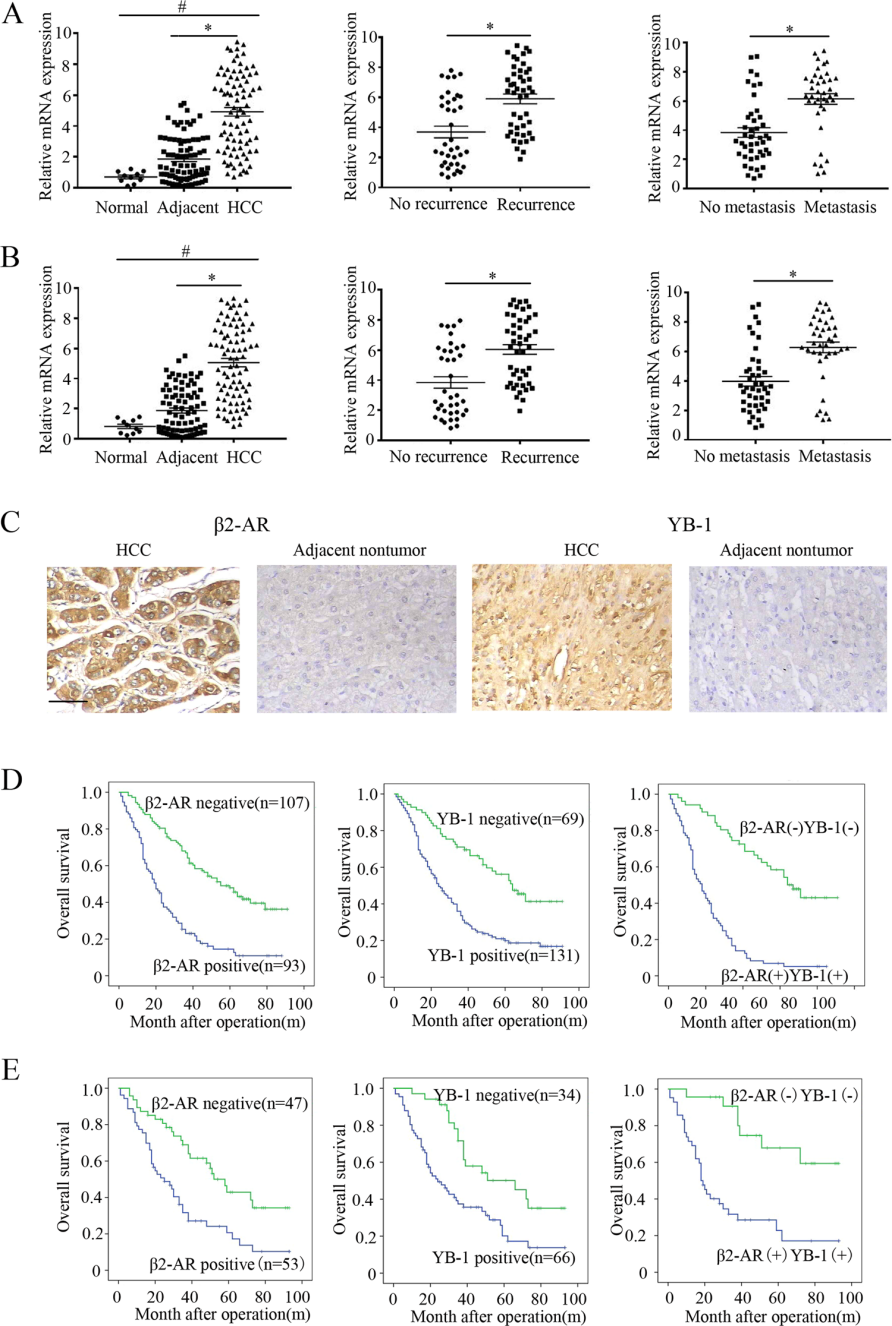
Clinicopathological correlation analysis of β2-AR and YB-1. **a** qRT-PCR analysis of β2-AR mRNA expression in HCC patients with recurrence (*n* = 46) or metastasis (*n* = 39). **b** qRT-PCR analysis of YB-1 mRNA expression in HCC patients with recurrence (*n* = 46) or metastasis (*n* = 39). **a**, **b** The data were mean ± SEM of three independent experiments. *P* value was determined by paired *t*-test(*^#^*P* < 0.05). **c** Representative pictures of IHC straining of β2-AR and YB-1 in human HCC tissues and the adjacent non-tumour tissues. Scale bar: 100 μm. **d** Kaplan–Meier analysis of the correlations between β2-AR and YB-1 protein level and overall survival of 200 patients with HCC (*P* < 0.05, log-rank test). **e** Kaplan–Meier analysis of the correlations between β2-AR and YB-1 protein level and overall survival of 100 patients with HCC (*P* < 0.05, log-rank test).

Many candidate β2-AR-interacting proteins (YB1, CPSM, MYH9, FLNA, LMNA, VIME, ENPL, CO4B, ANXA6, HS90A), among which YB-1 exhibited the greatest abundance and correlation with oncogenesis, were identified by using mass spectrometry. To understand the clinical relevance of YB-1 and β2-AR, we used two sets of HCC patient cohorts including 83 and 300 paired HCC tissues respectively. At first, we examined the mRNA level of β2-AR and YB-1 in 83 paired HCC samples and normal liver tissues (Fig. 1a, b). Compared to the expression in normal liver tissues, both non-tumour and tumour tissues showed higher expression of β2-AR and YB-1 in 83 paired HCC samples (Fig. 1a). The expression of β2-AR and YB-1 in HCC tissue is significantly higher than in non-tumour pairs. When we divided HCC tissues based on the recurrence or metastasis, the expression of β2-AR and YB-1 is significantly higher in recurrence or metastatic HCC tissues (Fig. 1a, b).

Next, we did immunohistochemistry and further analyzed the protein expression of β2-AR and YB-1 in 300 paired HCC tissues. In HCC tissues, β2-AR primarily localized in the cytoplasm, while YB-1 primarily localized in the nuclei (Fig. 1c). In addition, both proteins were abundant in HCC specimens compared with adjacent non-tumour tissues. Furthermore, the high expression of both β2-AR and YB-1 was correlated with tumour number, loss of tumour encapsulation, microvascular invasion, tumour differentiation, and higher TNM stage (Supplementary Table 1). Kaplan–Meier analysis showed that patients with increased expression of β2-AR or YB-1 had a statistically higher recurrence and metastasis rates and decreased OS (Fig. 1d, e and Supplementary Fig. 1A, B). Multivariate analysis indicated that both β2-AR and YB-1 were independent risk factors for recurrence and poor prognosis after curative HCC resection (Table 1 and Supplementary Table S4). Furthermore, β2-AR expression was positively correlated with YB-1 expression in HCC specimens. Kaplan–Meier analysis showed that patients with positive expression of both β2-AR and YB-1 had the highest recurrence and metastasis rates and the lowest OS (Fig. 1d, e and Supplementary Fig. 1). Taken together, these findings suggest that increased β2-AR and YB-1 expression were tightly associated with metastasis and an unfavourable prognosis in HCC patients.

**Table 1:** Univarate and multivariate analysis of factors associated with recurrence and survival in cohort I HCC patients (*n* = 200).

Variables	Recurrence						Survival					
	Univariate Analysis			Multivariate Analysis			Univariate Analysis			Multivariate Analysis		
	HR	95% CI	*P* Value	HR	95% CI	*P* Value	HR	95% CI	*P* Value	HR	95% CI	*P* Value
Age	0.752	0.514–1.098	0.140				0.804	0.576–1.121	0.198			
Sex (female versus male)	0.970	0.616–1.528	0.897				0.840	0.571–1.238	0.379			
Serum AFP (≤20 versus >20 ng/mL)	0.722	0.487–1.069	0.103				0.886	0.632–1.242	0.482			
HBV infection (no versus yes)	0.872	0.663–1.148	0.329				0.808	0.631–1.036	0.093			
Cirrhosis (absent versus present)	0.810	0.498–1.318	0.396									
Child-Pugh score (A versus B)	0.647	0.416–1.007	0.054				0.762	0.525–1.107	0.153			
Tumour number (single versus multiple)	0.646	0.437–0.953	0.028	1.344	0.860–2.101	0.195	0.593	0.422–0.834	0.003	1.188	0.803–1.756	0.389
Maximal tumour size (≤5 versus >5 cm)	0.484	0.291–0.807	0.005	0.590	0.348–1.001	0.050	0.587	0.365–0.946	0.029	0.676	0.413–1.107	0.119
Microvascular invasion (absent versus present)	0.644	0.426–0.974	0.037	0.401	0.257–0.624	<0.001	1.402	1.004–1.956	0.047	0.981	0.688–1.399	0.915
Tumour encapsulation (absent versus present)	1.421	0.955–2.115	0.083				1.459	1.027–2.072	0.035	0.964	0.661–1.405	0.849
Tumour differentiation (I–II versus III–IV)	0.330	0.223–0.488	<0.001	0.490	0.306–0.785	0.003	0.305	0.216–0.432	<0.001	0.474	0.309–0.728	0.001
TNM stage (I–II versus III)	0.421	0.286–0.619	<0.001	0.504	0.329–0.771	0.002	0.403	0.287–0.565	<0.001	0.617	0.422–0.902	0.013
β2-AR expression (negative versus positive)	0.370	0.251–0.546	<0.001	0.556	0.363–0.850	0.007	0.345	0.245–0.487	<0.001	0.584	0.397–0.861	0.007
YB-1 expression (negative versus positive)	0.452	0.295–0.694	<0.001	0.517	0.0324–0.826	0.006	0.402	0.274–0.590	<0.001	0.576	0.378–0.875	0.010

*HR* hazard ratio, *CI* confidence interval, *AFP* alpha-fetoprotein.

*P < 0.05.

### YB-1 binds to the cytoplasmic C-terminus of β2-AR

**Fig. 2:**
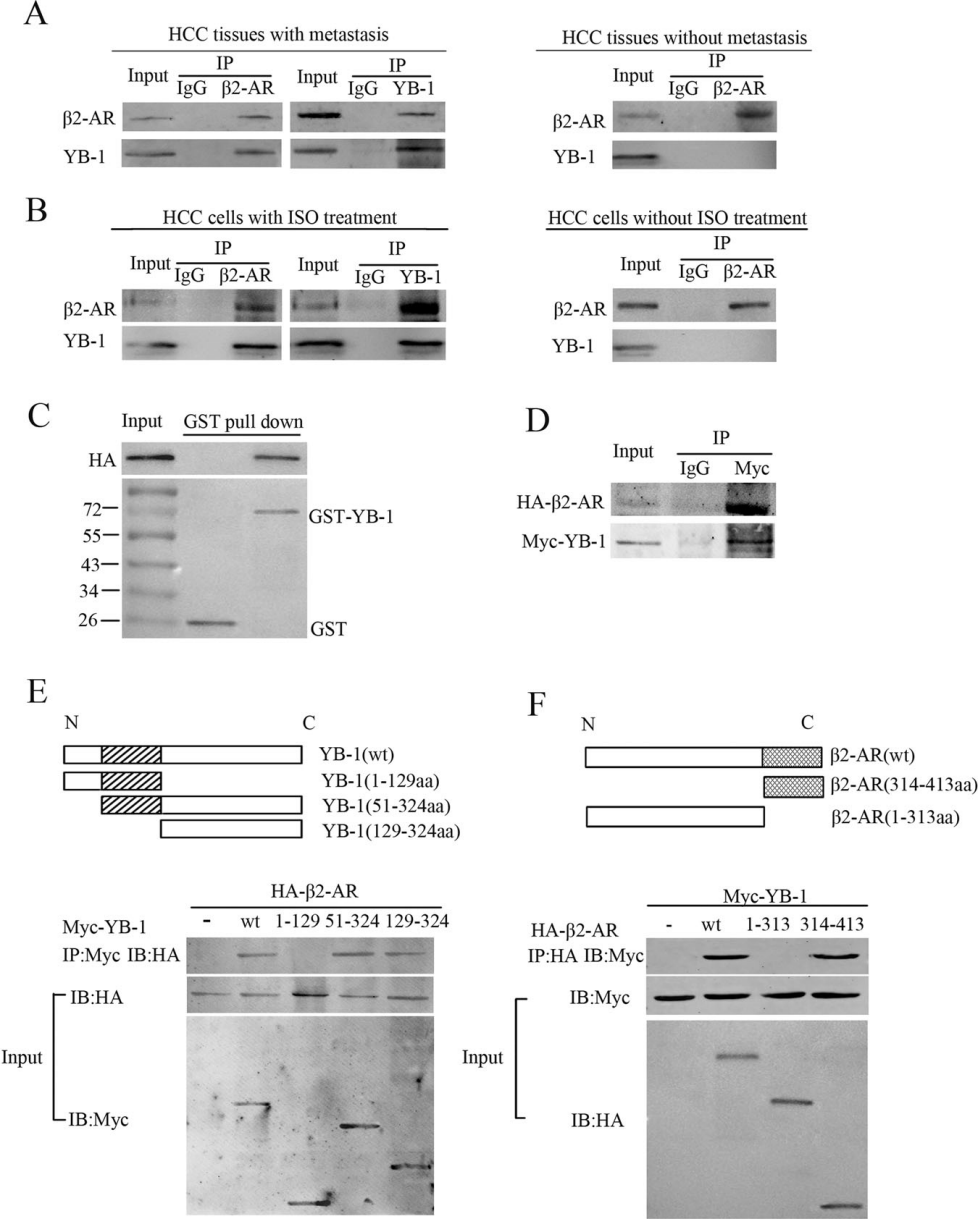
Assay for the interaction between β2-AR and YB-1. **a** Immunoprecipitation for β2-AR using anti-β2-AR and anti-YB-1 antibodies, respectively, in HCC specimens with metastasis (left, upper). Immunoprecipitation for YB-1 using anti-β2-AR and anti-YB-1 antibodies, respectively, in HCC specimens with metastasis (left, lower). Immunoprecipitation for β2-AR using anti-β2-AR and anti-YB-1 antibodies, respectively, in HCC specimens without metastasis (right, upper). Immunoprecipitation for YB-1 using anti-β2-AR and anti-YB-1 antibodies, respectively, in HCC specimens without metastasis (right, lower). **b** Immunoprecipitation for β2-AR using anti-β2-AR and anti-YB-1 antibodies, respectively, in SMMC-7721 cells pre-treated with 10 μM ISO (left, upper). Immunoprecipitation for YB-1 using anti-β2-AR and anti-YB-1 antibodies, respectively, in SMMC-7721 cells pre-treated with 10 μM ISO (Left, Lower). Immunoprecipitation for β2-AR using anti-β2-AR and anti-YB-1 antibodies, respectively, in SMMC-7721 cells without ISO treatment (right, upper). **c** Glutathione S-transferase (GST) pulldown of HA-β2-AR from whole-cell extracts of HEK293T cells transfected with GST or GST-YB-1 fusion proteins. **d** Association between ectopically expressed β2-AR and YB-1 in HEK293T cells. **e** Schematic diagram of full-length YB-1 and the generated deletion mutants. Co-IP for HA-β2-AR with YB-1 truncation mutants (upper). Western blotting of HA-β2-AR and YB-1 truncation mutants (middle and lower). **f** Schematic diagram of full-length β2-AR and the generated deletion mutants. Co-IP for Myc-YB-1 with β2-AR mutants (upper). Western blotting of Myc-YB-1 and β2-AR truncation mutants (middle and lower).

To gain better insight into the interaction between β2-AR and YB-1, co-immunoprecipitation (Co-IP) was performed in HCC tissue specimens and SMMC-7721 cells. As expected, YB-1 was pulled down in metastatic HCC specimens and SMMC-7721 cell lysates pre-treated with 10 μM ISO with an anti-β2-AR antibody, and β2-AR was pulled down with an anti-YB-1 antibody (Fig. 2a, b). These results reflect a physiological interaction between endogenous β2-AR and YB-1. A glutathione S-transferase (GST) pull-down assay showed that GST-YB-1, but not GST, co-precipitates with haemagglutinin (HA)-tagged β2-AR (Fig. 2c), suggesting that β2-AR interacts with YB-1 in vitro.

Vectors containing the sequences of full-length or truncated HA-tagged β2-AR and Myc-tagged YB-1 were subsequently constructed to identify the precise domain(s) responsible for the interaction between β2-AR and YB-1. HEK293T cells were transfected with these constructs, and Co-IP was performed to detect the potential interaction. Myc-tagged YB-1 immunoprecipitated with anti-HA antibody (Fig. 2d). Three different truncated YB-1 proteins (1–129 amino acids (aa), 51–324 aa, and 129–324 aa) and two truncated β2-AR proteins (1–313 aa and 314–413 aa) were constructed (Fig. 2e, f). Co-IP showed that full-length β2-AR interacts with both the 51–324 aa and 129–324 aa truncated YB-1 proteins, whereas full-length YB-1 interacts with the cytoplasmic C-terminus of β2-AR (314–413 aa) (Fig. 2e, f). These findings imply that β2-AR binds to specific domains of YB-1 in response to the stimulation of HCC cells with ISO.

### β2-AR facilitates YB-1 phosphorylation and nuclear translocation

**Fig. 3:**
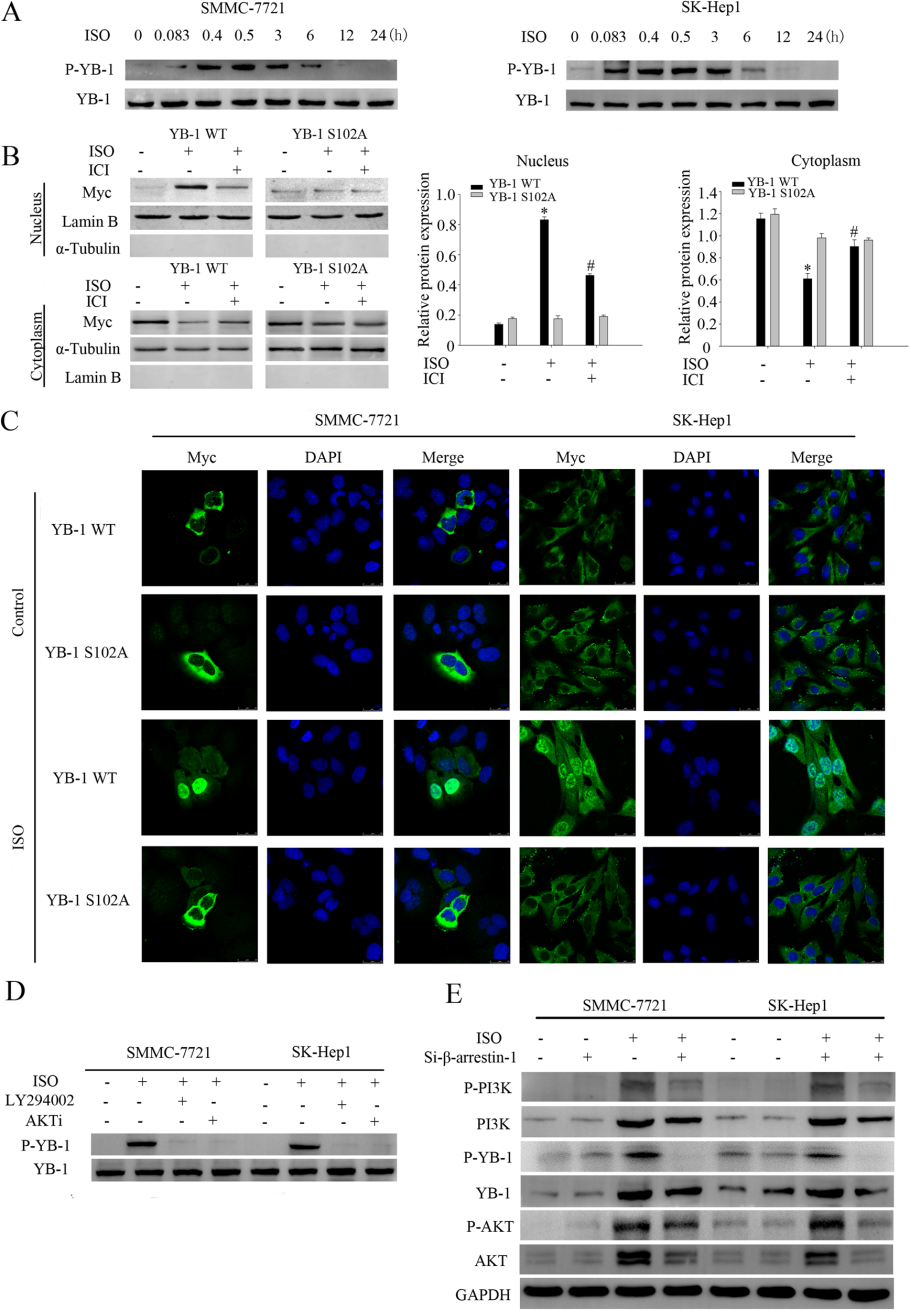
Determination of the phosphorylation and nuclear translocation of YB-1 in response to the stimulation of β2-AR. **a** Western blotting to detect total YB-1 and phosphorylated YB-1 (S102) proteins in HCC cells after ISO treatment for the indicated times. **b** Nuclear and cytoplasmic proteins were isolated from SMMC-7721 cells treated with 10 μM ISO for 6 h after transfection with YB-1 WT or YB-1 S102A for 48 h. The bar chart demonstrated the ratio of YB-1 expression to Lamin B/ɑ-Tubulin by densitometry. The data were mean ± SEM of three independent experiments (*^#^*P* < 0.05). **c** Confocal immunofluorescence microscopy showed that ISO treatment promotes the nuclear translocation of YB-1 but not YB-1 S102A in SK-Hep1 and SMMC-7721 cells. Scale bar: 25 μm. **d** Western blot analysis of phosphorylated YB-1 (S102) in SMMC-7721 and SK-Hep1 cells following treatment with ISO, LY294002 and AKTi. **e** HCC cells were transfected with control or β-arrestin-1-targeting siRNA and subsequently exposed to 10 μM ISO for 6 h, followed by western blot analysis.

To determine the underlying mechanism by which β2-AR regulates YB-1, the β-adrenoceptor agonist ISO was used to stimulate HCC cell lines. The addition of 10 μM ISO rapidly induced the phosphorylation of YB-1 (P-YB-1) at S102 in SMMC-7721 cells whereas β2-AR knockdown (Supplementary Fig. 1D) significantly attenuated these effects. This result was also observed in SK-Hep1 cells, another HCC cell line (Fig. 3a and Supplementary Fig. 1E). Interestingly, treatment with ISO efficiently promoted the entry of MYC-YB-1 into the nucleus, whereas a serine (S) to alanine (A) mutation at site 102 (S102A) abolished this nuclear translocation (Fig. 3b). Quantitative determination of the nuclear and cytoplasmic levels of the proteins validated the significantly increased nuclear protein levels of YB-1, but not YB-1 S102A, following ISO simulation, while the β2-AR antagonist ICI118,551 or β2-AR knockdown completely attenuated these effects (Fig. 3c and Supplementary Fig. 1F). These results demonstrate that β2-AR facilitates YB-1 phosphorylation and nuclear translocation in HCC cells.

### AKT and β-arrestin-1 mediate β2-AR-induced YB-1 phosphorylation

The PI3K/AKT cascade has been suggested to play a critical role for YB-1 phosphorylation at S102 site^27^, thus we tested whether β2-AR induced YB-1 phosphorylation also dependent on PI3K/AKT. To address this question, we applied PI3K inhibitor LY294002 and AKT inhibitor AKTi. Again, ISO treatment could strongly induce YB-1 phosphorylation, but YB-1 phosphorylation was almost completely inhibited by LY294002 and AKTi (Fig. 3d). β-arrestin-1 is a partner of AKT and also been showed to be critical in AKT mediated YB-1 phosphorylation. Therefore, we determined whether β-arrestin-1 is also important for β2-AR induced YB-1 phosphorylation. Knocking down β-arrestin-1 expression by siRNA attenuated the levels of phosphorylated YB-1 in ISO-treated cells (Fig. 3e). Collectively we demonstrated that β2-AR directly promotes YB-1 phosphorylation and nuclear translocation through a β-arrestin-1-mediated PI3K/AKT cascade. Interesting, P-YB-1 was pulled down in SMMC-7721 cell lysates pre-treated with 10 μM ISO with an anti-P-β2-AR antibody, and P-β2-AR was pulled down with an anti-P-YB-1 antibody (Supplementary Fig. 1C). Taken all these results together, there are possibly two pathways for YB-1 phosphorylation after ISO stimulation. One is direct interaction of β-AR and YB-1 and the other is β-arrestin-1 dependent pathway.

### β2-AR promotes epithelial-to-mesenchymal transition and the invasion of HCC cells by a YB-1-dependent mechanism

To evaluate the tumorigenic effects of the β2-AR-mediated phosphorylation of YB-1, the in vitro migration properties of normal hepatocytes (LO2) and HCC cell lines (LM3, SMMC-7721, HepG2, QSG-7701, PLC, SK-Hep1, and MHCC-97H) were determined. The expression of β2-AR and YB-1 at the transcriptional and translational levels was remarkably elevated, along with an increase in metastatic potential in HCC cell lines compared to that in normal hepatocytes (Supplementary Fig. 2A, B). Both SMMC-7721 and SK-Hep1 cells, which express high levels of β2-AR and YB-1, were further stimulated with ISO for 48 h in the presence or absence of ICI118,551. The application of ISO efficiently increased the migration and invasion of these two cell lines. However, pre-incubation with ICI118,551 or the knockdown of YB-1 significantly reduced these ISO-induced effects (Supplementary Fig. 2C, D). These data implicate the β2-AR-mediated phosphorylation of YB-1 as involved in the invasive phenotype in HCC cells.

**Fig. 4:**
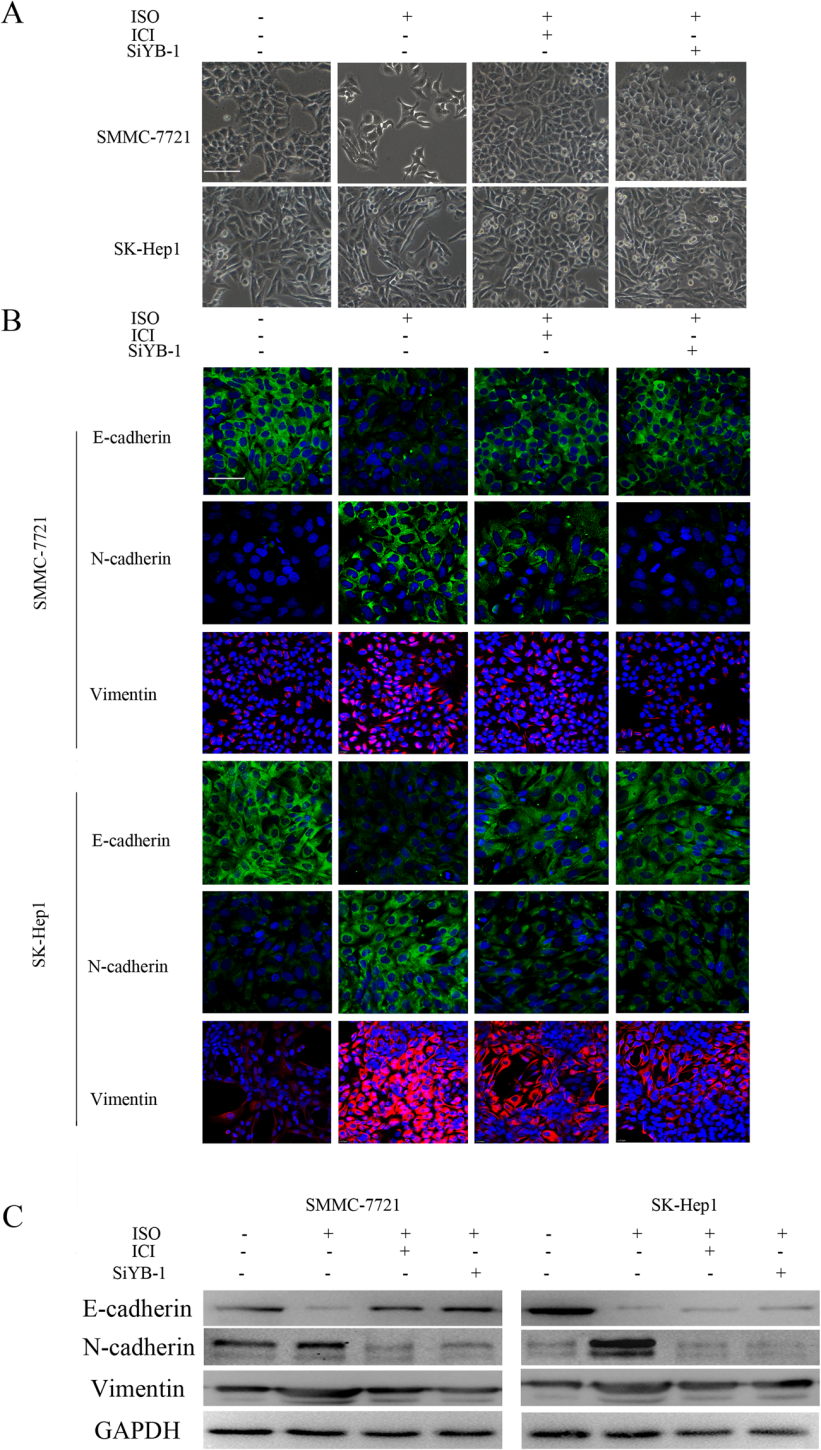
EMT assays in ISO-treated HCC cells. **a** Treatment with 10 μM ISO for 48 h affected the morphologies of SMMC-7721 and SK-Hep1 cells, while pre-incubation with 100 μM ICI118,551 or YB-1 knockdown inhibited the changes in cell morphology. Scale bar: 100 μm. **b** Immunofluorescence assay to detect the epithelial marker E-cadherin and the mesenchymal markers N-cadherin and vimentin in SMMC-7721 cells following the indicated treatments. Scale bar: 100 μm. **c** Western blot analysis to detect the expression of E-cadherin, N-cadherin and vimentin in SMMC-7721 and SK-Hep1 with different treatment.

Due to the appearance of a spindle-like mesenchymal morphology following the treatment of HCC cells with ISO, we assumed that the β2-AR-mediated activation of YB-1 elicits epithelial-to-mesenchymal transition (EMT), a key step in the metastasis of epithelial tumours, including HCC (Fig. 4a). Immunofluorescence staining showed that the β2-AR agonist ISO upregulates the expression of the mesenchymal markers N-cadherin and vimentin, whereas the reduced expression of the epithelial marker E-cadherin was observed following ISO treatment (Fig. 4b). Both pre-treatment of the cells with ICI118,551 and knockdown with YB-1 siRNA reversed the epithelial phenotype in SMMC-7721 and SK-Hep1 cells. Western blotting confirmed changes in the levels of EMT-related proteins (Fig. 4c). These data indicate that the β2-AR-mediated activation of YB-1 promotes EMT and the invasion of HCC cells.

### Phosphorylated YB-1 promotes the expression of β-catenin through binding to the β-catenin promoter

**Fig. 5:**
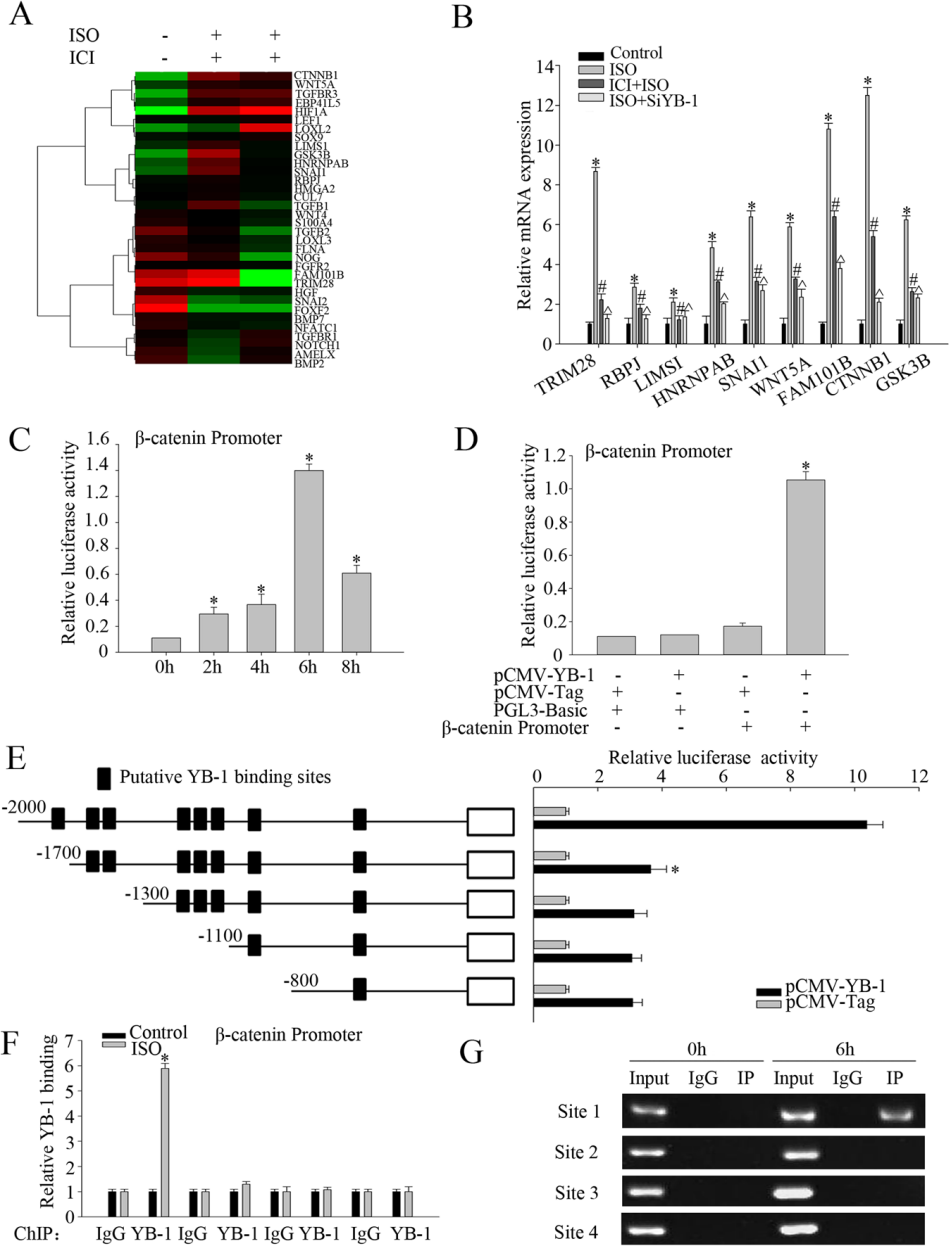
Analysis of YB-1-regulated target(s). **a** Two-dimensional hierarchical clustering of EMT-associated genes that are differentially expressed in SK-Hep1 cells and SK-Hep1 cells that had been transfected with YB-1 siRNA for 24 h were stimulated with 10 μM ISO for 6 h. Fold-changes in mRNA levels in ISO-treated cells with or without YB-1, with green and red squares indicating decreased and increased expression, respectively. **b** qRT-PCR analysis of the relative mRNA levels of nine EMT-associated genes in SK-Hep1 cells following the indicated treatments. The bar chart demonstrated the ratio of mRNA expression to GAPDH. The data were mean ± SEM of three independent experiments (*^#^^*P* < 0.05). **c** Transfection of β-catenin promoter and YB-1 full-length plasmid in SK-Hep1 cells with ISO treatment in different time points(2 h, 4 h, 6 h, 8 h). A luciferase reporter assay demonstrated a time-dependent increase in β-catenin promoter activity, which peaked at 6 h, following ISO treatment. The data were mean ± SEM of three independent experiments (*P < 0.05). **d** Transfection of pCMV-YB-1, pCMV-taq, PGL3-Basic and β-catenin promoter plasmids into SK-Hep1 cells with ISO stimulation for 6 h. A luciferase reporter assay was used to detect β-catenin promoter activity in different treatments. The data were mean ± SEM of three independent experiments (*P < 0.05). **e** Schematic diagram of putative YB-1-binding elements in the β-catenin promoter and the relative luciferase activities of different truncated β-catenin proteins with or without YB-1 overexpression. **f** Chromatin immunoprecipitation (ChIP) assay showing in vivo binding between YB-1 and β-catenin promoter regions in ISO-treated SK-Hep1 cells. The use of anti-YB-1 antibody following the ChIP assay with anti-YB-1 antibody allowed the detection of the resultant DNA samples using real-time PCR. The data were mean ± SEM of three independent experiments (*P < 0.05). **g** Representative images of semi-quantitative PCR analysis of YB-1-bound β-catenin promoter fragments in ISO-treated SK-Hep1 cells.

Phosphorylated YB-1 shuttles from the cytoplasm to the nucleus, where it acts as a transcription factor to induce the expression of oncogenes. To identify the key target(s) of nuclear YB-1, SK-Hep1 cells and SK-Hep1 cells that had been transfected with YB-1 siRNA for 24 h were stimulated with 10 μM ISO for 6 h. Microarray analysis was performed to compare the transcriptional profiles of SK-Hep1 cells subjected to YB-1 knockdown. A total of 18,893 differentially expressed genes (DEGs) in the three groups (mock-, ISO-, and ISO-treated with YB-1 siRNA knockdown) were identified. Gene ontology (GO) analysis revealed 34 DEGs related to EMT, the expression levels of which were dynamic, as shown by hierarchical cluster analysis (Fig. 5a). Among these DEGs, 9 genes (*Trim28*, *rbpj*, *lims1*, *hnrnpab*, *snail*, *wnt5a*, *fam101b*, *ctnnb1*, and *gsk3b*) responded to stimulation with ISO and YB-1 depletion, suggesting that they are target(s) of the YB-1 protein (Fig. 5a, b). Specifically, *ctnnb1*, which encodes the β-catenin protein, exhibited the most abundant expression, and the difference in its expression was the most significant (Fig. 5b). Considering its critical role in triggering EMT in various cancer types, including HCC, β-catenin was therefore screened for further validation.

**Fig. 6:**
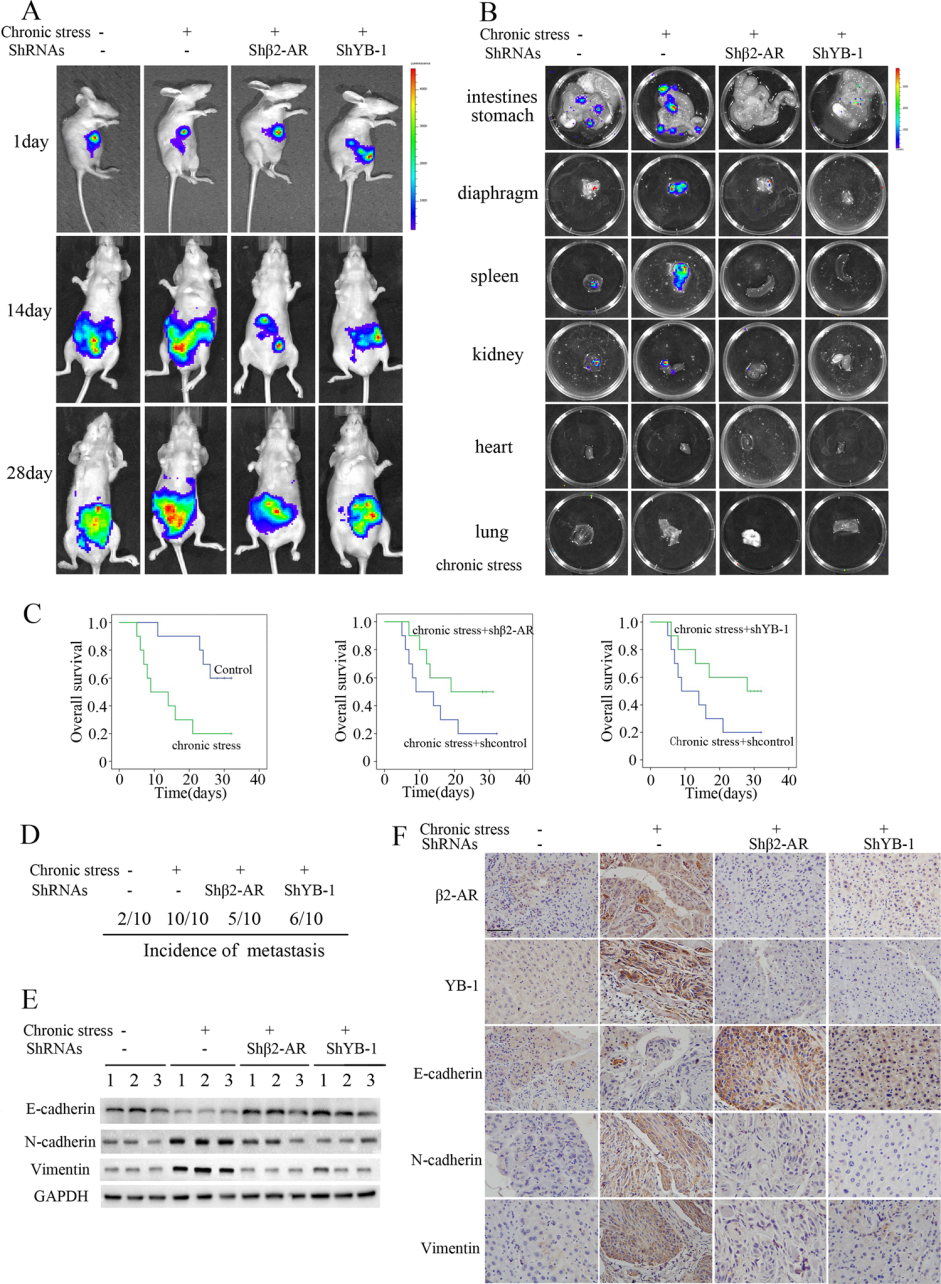
β2-AR/YB-1 mediates the progression of HCC. **a** Following the injection of stable lentiviral-infected HCC cells, the tumours derived from SK-Hep1 cells at liver implantation sites in mice in different groups were monitored. Here are the representative images. **b**, **d** The incidence of organ metastases was higher in mice implanted with SK-Hep1-Lv-shcontrol than in other groups of mice in the presence of chronic stress. **c** Kaplan–Meier analysis of nude mice that received the indicated orthotropic liver implantations (*P* < 0.05, log-rank test). **e** Western blot analysis to detect the expression of E-cadherin, N-cadherin, and vimentin in different groups. **f** IHC analysis to detect the expression of E-cadherin, N-cadherin and vimentin in HCC tissue specimens from nude mice. Scale bar: 100 μm.

The putative Y-box elements in the β-catenin promoter region were cloned into pRL-CMV vectors, which were used to transfect SK-Hep1 cells. The cells were stimulated with ISO for 48 h. A luciferase reporter assay demonstrated a time-dependent increase in β-catenin promoter activity, which peaked at 6 h, following ISO treatment (Figs. 5c and 6d). Deletion of the −2000 to −1700 region of the β-catenin promoter significantly impaired the effects of YB-1; however, the deletion of a region from −1700 to −800 had no effect (Fig. 5e). We further designed four pairs of primers based on the following sites: Site 1 (−1853), Site 2 (−1593, −1580), Site 3 (−1269, −1252, −1193), and Site 4 (−1071, −783). The results of ChIP assays in ISO-induced SK-Hep1 cells were consistent (Fig. 5f, g) and indicated the β-catenin promoter sequence from −2000 to −1700 as a critical binding site of YB-1.

### Chronic stress promotes HCC metastasis via the β2-AR/YB-1 axis in vivo

In the context of chronic stress, catecholamines are released by the neuroendocrine system to modulate carcinogenesis. To determine whether HCC metastasis is modulated by chronic stress via β2-AR/YB-1 signalling, a restraint-stress model utilizing a physical restraint system for 2 h each day was designed. Orthotopic xenografts were produced by injecting HCC cells into mouse livers to establish mouse liver tumour models. Mice subjected to chronic restraint stress for 4 weeks exhibited rapid tumour growth and metastasis (Fig. 6a). However, the knockdown of β2-AR or YB-1 expression through the injection of 50 μL of luciferase-expressing siRNA lentivirus significantly suppressed the effects of chronic stress (Fig. 6a). Chronic stress contributed to the incidence of organ metastases and a reduction in OS, which were reversed by β2-AR or YB-1 interference (Fig. 6b, c). Western blotting and immunostaining demonstrated a remarkable increase in the expression of β2-AR, YB-1, N-cadherin and vimentin and decrease in the expression of E-cadherin, which are governed by the β2-AR/YB-1 axis, in liver cancer tissues from mice subjected to chronic stress (Fig. 6e, f). These findings indicate that the β2-AR/YB-1 axis is a key modulator of chronic stress-induced HCC metastasis.

## Discussion

Since 1926, psychosocial stress has been closely associated with carcinogenesis^28^. This led to the consensus that tumour cell migration and invasion are not only genetically determined but also distinctly environmentally regulated^29^. Long-term anxiety in patients with HCC triggers adrenergic signalling, which worsens prognosis^11,30^. As an important receptor of stress-induced catecholamines, β2-AR is implicated in tumour development, growth, and progression, including migration. Currently, the exact mechanism linking β2-AR activation to malignant progression is unclear. Here, we identified a novel β2-AR/YB-1/β-catenin axis that participated in the regulation of HCC metastasis in response to chronic stress. Our results provide a new avenue for the development of β-blocker-based therapy for HCC metastasis.

β2-AR is one of nine ARs (α_1_A, α_1_B, α_1_D, α_2_A, α_2_B, α_2_C, β1, β2 and β3) that transduce the effects of (nor)epinephrine in a variety of cell types^31^. Signalling from β2-AR is first initiated by either a G protein or β-arrestin. β2-AR agonist triggers the coupling of β2-AR to Gs, activating either cAMP-dependent protein kinase A (PKA) or guanine exchange proteins directly activated by cAMP (EPACs). PKA subsequently either activates cytoplasmic kinases, including mitogen-activated protein kinase (MAPK)^32^, or enters the nucleus to phosphorylate the CREB transcription factor^11^. PKA can also mediate phosphorylation of β2-AR, causing it to couple with Gi. This G protein switching is associated with the Gbγ-mediated activation of alternative signal pathways, such as the PI3K/AKT pathway^33^. EPACs further transduce signals through the Rap family of small Ras-like GTPases^34^. Alternatively, β2-AR can induce cellular responses that involve members of the β-arrestin protein family. β-Arrestins not only participate in the attenuation of β2-AR signalling and receptor internalisation but also play roles in signal transduction by their connection to multiple signalling pathways, such as the p38, ERK1/2 and NF-κB signalling pathways^35^. In the present study, we have shown that the β2-AR agonist ISO can facilitate the phosphorylation of YB-1 in an AKT activity-dependent manner in HCC cells. Meanwhile, β-arrestin was shown to be an indispensable signal transducer rather than a signalling inhibitor. These findings suggest that the synergistic effects of β2-AR-mediated pathways are involved in the progression of HCC.

YB-1 is implicated in the malignant phenotypes of various carcinomas. Cytoplasmic YB-1 facilitates the translational activation of Snail, HIF1α and Twist, which induces EMT in cancer cells^36,37^. A recent study showed that nuclear YB-1 also facilitates TGF-β-induced EMT, suggesting the transcriptional regulation of EMT^38^. Pharmacological inhibition of YB-1 phosphorylation at S102 abrogates the EGF-induced expression of EMT markers^39^, demonstrating that YB-1 phosphorylation at S102 is critical for its pro-tumorigenic activity. In the present study, we performed transcriptional profile analysis and ChIP assays to identify the downstream target(s) of YB-1. The critical and multi-functional protein β-catenin, in addition to other EMT-promoting factors including Snail and Twist, was shown to be regulated by YB-1 in response to the stimulation of β2-AR in HCC cells. It is reported that knockdown of YB-1 impairs Wnt/β-catenin signalling pathway and reduces the numbers of HCC initiating cells, whereas YB-1 also mediates breast cancer invasion and metastasis via regulation of MMP1 and β-catenin^40,41^. Such findings might be helpful to address the complex cellular events during HCC metastasis and provide a potential target for pharmaceutical development.

## Conclusion

In the context of chronic stress, β2-AR signalling induces the phosphorylation of YB-1 at S102 via β-arrestin-1-dependent activation of the PI3K/AKT pathway. Activation of YB-1 promotes the expression of β-catenin, which mediates the progression of HCC.

## Supplementary information

Supplementary Tables

Supplementary Figure 1

Supplementary Figure 2

Supplementary Figure legends
