# Supplementary material for: A novel β2-AR/YB-1/β-catenin axis mediates chronic stress-associated metastasis in hepatocellular carcinoma
Source: Oncogenesis. 2020 Sep 24;9(9):84. doi: 10.1038/s41389-020-00268-w (PMC7515897; doi:10.1038/s41389-020-00268-w)
Supplement: Supplementary file 1 — Supplementary Tables [file 41389_2020_268_MOESM1_ESM.docx]

**Table S1. Correlation Between β2-AR and YB-1 Expression and Clinicopathological Characteristics of HCCs in two independent cohorts of human HCC tissues**

|  | | **Cohort I** | | | | | | **Cohort II** | | | | | |
| --- | --- | --- | --- | --- | --- | --- | --- | --- | --- | --- | --- | --- | --- |
|  |  | **β2-AR expression** | |  | **YB-1 expression** | |  | **β2-AR expression** | |  | **YB-1 expression** | |  |
| **Clinicopathological**  **Variables** | | **Negative**  **(n=107 )** | **Positive**  **(n=93)** | ***P* Value** | **Negative**  **(n=69 )** | **Positive**  **(n=131)** | ***P* Value** | **Negative**  **(n=47 )** | **Positive**  **(n=53)** | ***P* Value** | **Negative**  **(n= 34)** | **Positive**  **(n=66)** | ***P* Value** |
| Age |  | 47.6 | 51.7 | 0.119 | 48.9 | 49.8 | 0.457 | 53.8 | 47.6 | 0.822 | 54.5 | 48.4 | 0.478 |
| Sex | Female | 25 | 22 | 0.961 | 15 | 32 | 0.670 | 8 | 10 | 0.810 | 6 | 12 | 0.947 |
|  | Male | 82 | 71 |  | 54 | 99 |  | 39 | 43 |  | 28 | 54 |  |
| Serum AFP | ≤20 ng/mL | 44 | 38 | 0.970 | 30 | 52 | 0.605 | 23 | 35 | 0.084 | 18 | 40 | 0.462 |
|  | >20 ng/mL | 63 | 55 |  | 39 | 79 |  | 24 | 18 |  | 16 | 26 |  |
| Virus infection | HBV | 76 | 62 | 0.664 | 48 | 90 | 0.191 | 34 | 40 | 0.944 | 26 | 48 | 0.752 |
|  | HCV | 6 | 4 |  | 6 | 4 |  | 1 | 1 |  | 0 | 2 |  |
|  | HBV+HCV | 7 | 5 |  | 5 | 7 |  | 2 | 3 |  | 2 | 3 |  |
|  | None | 18 | 22 |  | 10 | 30 |  | 10 | 9 |  | 6 | 13 |  |
| Cirrhosis | Absent | 24 | 15 | 0.262 | 17 | 22 | 0.183 | 17 | 19 | 0.973 | 10 | 26 | 0.325 |
|  | Present | 83 | 78 |  | 52 | 109 |  | 30 | 34 |  | 24 | 40 |  |
| Child-Pugh score | Class A | 68 | 72 | 0.033* | 42 | 98 | 0.041* | 34 | 45 | 0.124 | 25 | 54 | 0.335 |
|  | Class B | 39 | 21 |  | 27 | 33 |  | 13 | 8 |  | 9 | 12 |  |
| Tumor number | Single | 83 | 46 | <0.001* | 52 | 77 | 0.020* | 37 | 29 | 0.011* | 27 | 39 | 0.042* |
|  | Multiple | 24 | 47 |  | 17 | 54 |  | 10 | 24 |  | 7 | 27 |  |
| Maximal tumor size | ≤ 5 cm | 98 | 77 | 0.061 | 62 | 113 | 0.465 | 36 | 35 | 0.246 | 26 | 45 | 0.387 |
|  | > 5 cm | 9 | 16 |  | 7 | 18 |  | 11 | 18 |  | 8 | 21 |  |
| Tumor encapsulation | Absent | 57 | 64 | 0.025* | 35 | 86 | 0.040* | 14 | 30 | 0.007* | 8 | 36 | 0.003* |
|  | Present | 50 | 29 |  | 34 | 45 |  | 33 | 23 |  | 26 | 30 |  |
| Microvascular invasion | Absent | 76 | 43 | <0.001* | 49 | 70 | 0.016* | 23 | 36 | 0.054 | 17 | 42 | 0.189 |
|  | Present | 31 | 50 |  | 20 | 61 |  | 24 | 17 |  | 17 | 24 |  |
| Tumor differentiation | I-II | 85 | 29 | <0.001* | 55 | 59 | <0.001* | 33 | 24 | 0.012* | 26 | 31 | 0.005* |
|  | III-IV | 22 | 64 |  | 14 | 72 |  | 14 | 29 |  | 8 | 35 |  |
| TNM stage | I-II | 81 | 30 | <0.001* | 50 | 61 | <0.001* | 41 | 29 | <0.001* | 30 | 40 | 0.004* |
|  | III | 26 | 63 |  | 19 | 70 |  | 6 | 24 |  | 4 | 26 |  |

Abbreviation: AFP, alpha-fetoprotein

**P* < 0.05

**Table S2. Primary antibodies for Western blot analysis, immunohistochemistry, co-immunoprecipitation, immunofluorescence assay.**

| **Antibody** | **WB** | **IHC** | **Co-IP** | **IF** | **Specificity** | **Number** | **Company** |
| --- | --- | --- | --- | --- | --- | --- | --- |
| β2-AR | + | + | + | + | polyclonal | Sc-9042 | Santa Cruz |
| YB-1 | + | + | + | + | polyclonal | Ab12148 | Abcam |
| HA | + | - | + | - | polyclonal | Ab9110 | Abcam |
| Myc  GST | +  + | -  - | +  - | -  - | Polyclonal  monoclonal | Ab9106  Sc-53909 | Abcam  Santa Cruz |
| P-YB-1^Ser102^ | + | - | - | - | polyclonal | Ab138654 | Abcam |
| P-β2-AR | + | + | + | + | polyclonal | Ab62464 | Abcam |
| α-Tubulin | + | - | - | - | polyclonal | Ab18251 | Abcam |
| Lamin B | + | - | - | - | monoclonal | Sc-56143 | Santa Cruz |
| β-arrestin-1 | + | - | - | - | polyclonal | Ab31868 | Abcam |
| P-PI3K | + | - | - | - | polyclonal | 4228 | Cell Signaling |
| P-AKT | + | - | - | - | monoclonal | 4060 | Cell Signaling |
| GAPDH | + | - | - | - | monoclonal | Ab9484 | Abcam |
| E-cadherin | + | + | - | + | monoclonal | 3195 | Cell Signaling |
| N-cadherin | + | + | - | + | monoclonal | 13116 | Cell Signaling |
| Vimentin | + | + | - | + | monoclonal | 5741 | Cell Signaling |
| β-catenin | + | - | - | - | monoclonal | 8480 | Cell Signaling |

**Table S3. Primer sequences used in the study.**

| **Primer name** | **Primer sequences** | **Enzyme** |
| --- | --- | --- |
| Primers for real-time PCR: |  |  |
| β2-AR sense: | 5'- CTTGCTGGCACCCAATAGAA -3' |  |
| β2-AR antisense: | 5'-TGATGAAGTAGTTGGTGACCGTC-3' |  |
| YB-1 sense: | 5'- AGAAATGAACAAAAGATTGGAGC -3' |  |
| YB-1 antisense: | 5'- AAGGCGTATTGAGAAAAACCAG -3' |  |
| β-catenin sense: | 5'-GAAGAGGCTGAAGGCAAAG-3' |  |
| β-catenin antisense: | 5'-AGCAGAGGGAATCAAACA-3' |  |
| WNT5A sense: | 5'- AGGGCTCCTACGAGAGTGCT -3' |  |
| WNT5A antisense: | 5'- GACACCCCATGGCACTTG -3' |  |
| TRIM28 sense: | 5'- GGGCTCTGGAGAGTGACAAC -3' |  |
| TRIM28 antisense: | 5'- GCAGGGCCTGTTGAGTTAGT -3' |  |
| SNAI1 sense: | 5'-TTACCTTCCAGCAGCCCTAC-3' |  |
| SNAI1 antisense: | 5'-AGCCTTTCCCACTGTCCTC-3' |  |
| LIMS1 sense: | 5'- TGGAGAGCTTAGCTTGGACAC -3' |  |
| LIMS1 antisense: | 5'- TTCACGATCTTCTCAGCGGG -3' |  |
| RBPJ sense: | 5'- CTGGATCCATGCAACCTGGC -3' |  |
| RBPJ antisense: | 5'- CAACTCGAGGGACACTACTGCTGC -3' |  |
| HNRNPAB sense: | 5'- TTTGGCGAGTTTGGGGAGATT -3' |  |
| HNRNPAB antisense: | 5'- GCCATACTGCTGCTGCTGATAGAC -3' |  |
| FAM101B sense: | 5'-GGCGAAGGAGTGGAGTTTGA-3' |  |
| FAM101B antisense: | 5'-CGGCAGTTGTAATCCAGGGT-3' |  |
| GSK3B sense: | 5'-GTGACAACAGTGGTGGCAAC-3' |  |
| GSK3B antisense: | 5'-CGGAACATAGTCCAGCACCA-3' |  |
| GAPDH sense: | 5’-GCACCGTCAAGGCTGAGAAC-3’ |  |
| GAPDH antisense: | 5’-TGGTGAAGACGCCAGTGGA-3’ |  |
| Primers for β-catenin promoter construct: |  |  |
| pGL3-β-catenin sense: | 5’-CGGGGTACCTCAGTAGGGATTAAAAATCA-3‘ | KpnI |
| pGL3-β-catenin antisense: | 5’-CCGCTCGAGGCCGCCACCGGCGCAGCGC-3‘ | XhoI |
| (-1700/0)snail sense: | 5’-CGGGGTACCTGACAATTAAAATTAGGAA-3‘ | KpnI |
| (-1300/0)snail sense: | 5’-CGGGGTACCGTTTTCCGCCTGCATCTATG-3‘ | KpnI |
| (-1100/0)snail sense: | 5’-CGGGGTACCGCATTAGAATGGGAAACAT-3‘ | KpnI |
| (-800/0)snail sense: | 5’-CGGGGTACCTCCTAAGGACTTGTTGAATT-3‘ | KpnI |
| antisense: | 5’-CCGCTCGAGGCCGCCACCGGCGCAGCGC-3‘ | XhoI |
| Primers for YB-1 Ser102A mutagenesis: |  |  |
| Ser102A mutation sense: | 5’-ACCTTCGCGCAGTAGGAGATGGAGAGACT-3’ |  |
| Ser102A mutation antisense: | 5‘-CTCCTACTGCGCGAAGGTACTTCCTGGGG-3’ |  |
| Primers used for ChIP in the β-catenin promoter: |  |  |
| β-catenin binding site 1 sense: | 5’- TTATTTTGGTAATGGAACAGAG-3’ |  |
| β-catenin binding site 1 antisense: | 5’- TACATTGAAAATCAGACGACA-3’ |  |
| β-catenin binding site 2 sense: | 5’- GGTAACTTTCACTGCTGCTTT-3’ |  |
| β-catenin binding site 2 antisense: | 5’- CTCTAGGCTATTGTTTGTGCTT-3’ |  |
| β-catenin binding site 3 sense: | 5’- ACGGTTTTGATGAAATACCTT-3’ |  |
| β-catenin binding site 3 antisense: | 5’- ATGTTCAGTGAGATCCATTGTC-3’ |  |
| β-catenin binding site 4 sense: | 5’- CCTAGTGACAAGTGGAACCAGA-3’ |  |
| β-catenin binding site 4 antisense: | 5’- CCTACTGTGAACAAATAACTAATGG -3’ |  |
| Primers for β2-AR sequential truncations: |  |  |
| (1-313aa) β2-AR sense: | 5’-CGGATCCGCCACCATGTACCCATACGATGTTCCAGATTACGCTGGGCAACCCGGGAACGGC -3’ | BamHI |
| (1-313aa) β2-AR antisense: | 5’-CCTCTAGATTAAGTCCACATTTTCAT -3’ | BamHI |
| (314-413aa) β2-AR sense: | 5’-CGGATCCGCCACCATGTACCCATACGATGTTCCAGATTACGCTATAGGCTATGTCAATTCT -3’ | BamHI |
| (314-413aa) β2-AR antisense: | 5’-CCTCTAGATTACAGCAGTGAGTCAT -3’ | BamHI |
| Primers for YB-1 sequential truncations: |  |  |
| (1-129aa)YB-1 sense: | 5’-CGGATCCGCCACCATGGAACAAAAACTCATCTCAGAAGAGGATCTGAGCAGCGAGGCCGAGACC-3’ | BamHI |
| (1-129aa)YB-1 antisense: | 5’-CCTCTAGATTAACCAGGACCTGTAAC-3’ | BamHI |
| (51-324aa)YB-1 sense: | 5’-CGGATCCGCCACCATGGAACAAAAACTCATCTCAGAAGAGGATCTGGACAAGAAGGTCATCGCA-3’ | BamHI |
| (51-324aa)YB-1 antisense: | 5’-CCTCTAGATTACTCAGCCCCGCCCTGCT-3’ | BamHI |
| (129-324aa)YB-1 sense: | 5’-CGGATCCGCCACCATGGAACAAAAACTCATCTCAGAAGAGGATCTGGGTGGTGTTCCAGTTCAA-3’ | BamHI |
| (129-324aa)YB-1 antisense: | 5’-CCTCTAGATTACTCAGCCCCGCCCTGCT-3’ | BamHI |
| Primers for pcDNA6.2-EGFP-β2-AR: |  |  |
| β2-AR-1#-miRNA sense: | 5’-TGCTGCACACCATCAGAATGATCACCGTTTTGGCCACTGACTGACGGTGATCACTGATGGTGTG-3’ |  |
| β2-AR-1#-miRNA antisense: | 5’-CCTGCACACCATCAGTGATCACCGTCAGTCAGTGGCCAAAACGGTGATCATTCTGATGGTGTGC-3’ |  |
| β2-AR-2#-miRNA sense: | 5’-TGCTGTAGACGAAGACCATGATCACCGTTTTGGCCACTGACTGACGGTGATCAGTCTTCGTCTA-3’ |  |
| β2-AR-2#-miRNA antisense: | 5’-CCTGTAGACGAAGACTGATCACCGTCAGTCAGTGGCCAAAACGGTGATCATGGTCTTCGTCTAC-3’ |  |
| β2-AR-3#-miRNA sense: | 5’-TGCTGTTAGGAGGATGTAAACTTCCTGTTTTGGCCACTGACTGACAGGAAGTTCATCCTCCTAA-3’ |  |
| β2-AR-3#-miRNA antisense: | 5’-CCTGTTAGGAGGATGAACTTCCTGTCAGTCAGTGGCCAAAACAGGAAGTTTACATCCTCCTAAC-3’ |  |
| Primers for pcDNA6.2-EGFP-YB-1: |  |  |
| YB-1-1#-miRNA sense: | 5’-TGCTGACAACATCAAACTCCACAGTCGTTTTGGCCACTGACTGACGACTGTGGTTTGATGTTGT-3’ |  |
| YB-1-1#-miRNA antisense: | 5’-CCTGACAACATCAAACCACAGTCGTCAGTCAGTGGCCAAAACGACTGTGGAGTTTGATGTTGTC-3’ |  |
| YB-1-2#-miRNA sense: | 5’-TGCTGTGGATAGCGTCTATAATGGTTGTTTTGGCCACTGACTGACAACCATTAGACGCTATCCA-3’ |  |
| YB-1-2#-miRNA antisense: | 5’- CCTGTGGATAGCGTCTAATGGTTGTCAGTCAGTGGCCAAAACAACCATTATAGACGCTATCCAC-3’ |  |
| YB-1-3#-miRNA sense: | 5’-TGCTGTATTCTGCCTCACTGGTCTACGTTTTGGCCACTGACTGACGTAGACCAGAGGCAGAATA-3’ |  |
| YB-1-3#-miRNA antisense: | 5’-CCTGTATTCTGCCTCTGGTCTACGTCAGTCAGTGGCCAAAACGTAGACCAGTGAGGCAGAATAC-3’ |  |
| Primers for pLenti6.3-β2-AR-EGFP-miR: |  |  |
| β2-AR sense: | 5'-TACTGGCGCGCCGCCACCATGGTGAGCAAGGGCGAGGA-3' | Asc1 |
| β2-AR antisense: | 5'-ACTAGTTTAAACTGCGGCCAGATCTGGGC-3' | Pme1 |
| Primers for pLenti6.3-YB-1-EGFP-miR: |  |  |
| YB-1 sense: | 5'-TACTGGCGCGCCGCCACCATGGTGAGCAAGGGCGAGGA-3' | Asc1 |
| YB-1 antisense: | 5'-ACTAGTTTAAACTGCGGCCAGATCTGGGC-3' | Pme1 |

**Table S4. Univarate and Multivariate Analysis of Factors Associated With Recurrence and Survival in Cohort II HCC patients (n = 100)**

|  | **Recurrence** | | | | | | **Survival** | | | | | |
| --- | --- | --- | --- | --- | --- | --- | --- | --- | --- | --- | --- | --- |
| **Variables** | **Univariate Analysis** | | | **Multivariate Analysis** | | | **Univariate Analysis** | | | **Multivariate Analysis** | | |
|  | **HR** | **95% CI** | ***P* Value** | **HR** | **95% CI** | ***P* Value** | **HR** | **95% CI** | ***P* Value** | **HR** | **95% CI** | ***P* Value** |
| Age | 1.155 | 0.707-1.890 | 0.565 |  |  |  | 0.993 | 0.615-1.605 | 0.978 |  |  |  |
| Sex (female versus male) | 0.943 | 0.502-1.769 | 0.854 |  |  |  | 1.137 | 0.579-2.232 | 0.710 |  |  |  |
| Serum AFP (≤20 versus >20 ng/mL) | 0.870 | 0.528-1.435 | 0.586 |  |  |  | 0.832 | 0.510-1.359 | 0.463 |  |  |  |
| HBV infection (no versus yes) | 0.854 | 0.455-1.603 | 0.622 |  |  |  | 0.902 | 0.491-1.660 | 0.741 |  |  |  |
| Cirrhosis (absent versus present) | 0.795 | 0.481-1.312 | 0.369 |  |  |  | 0.903 | 0.552-1.478 | 0.685 |  |  |  |
| Child-Pugh score (A versus B) | 0.574 | 0.298-1.105 | 0.097 |  |  |  | 0.591 | 0.314-1.113 | 0.103 |  |  |  |
| Tumor number (single versus multiple) | 0.384 | 0.232-0.634 | <0.001* | 0.984 | 0.530-1.827 | 0.959 | 0.345 | 0.213-0.559 | <0.001* | 0.854 | 0.467-1.562 | 0.609 |
| Maximal tumor size (≤5 versus >5 cm) | 0.581 | 0.346-0.975 | 0.040* | 0.724 | 0.411-1.276 | 0.264 | 0.581 | .351-0.961 | 0.034* | 0.742 | 0.429-1.284 | 0.286 |
| Microvascular invasion (absent versus present) | 0.576 | 0.352-0.942 | 0.028* | 0.549 | 0.288-1.046 | 0.068 | 0.603 | 0.373-0.977 | 0.040* | 0.578 | 0.312-1.073 | 0.083 |
| Tumor encapsulation (absent versus present) | 1.788 | 1.093-2.924 | 0.021* | 1.142 | 0.674-1.937 | 0.622 | 1.736 | 1.074-2.806 | 0.024* | 1.144 | 0.680-1.924 | 0.612 |
| Tumor differentiation (I-II versus III-IV) | 0.277 | 0.163-0.470 | <0.001* | 0.519 | 0.277-0.971 | 0.040* | 0.269 | 0.161-0.449 | <0.001* | 0.501 | 0.275-0.915 | 0.025* |
| TNM stage (I-II versus III) | 0.263 | 0.156-0.444 | <0.001* | 0.510 | 0.276-0.943 | 0.032* | 0.261 | 0.159-0.428 | <0.001* | 0.487 | 0.271-0.874 | 0.016* |
| β2-AR expression (negative versus positive) | 0.410 | 0.245-0.686 | 0.001* | 0.489 | 0.264-0.907 | .0.023* | 0.420 | 0.254-0.694 | 0.001* | 0.520 | 0.283-0.955 | 0.035* |
| YB-1 expression (negative versus positive) | 0.431 | 0.249-0.748 | 0.003* | 0.514 | 0.277-0.955 | 0.035* | 0.420 | 0.243-0.724 | 0.002* | 0.512 | 0.274-0.956 | 0.036* |

Abbreviations: HR, hazard ratio; CI, confidence interval; AFP, alpha-fetoprotein.

**P* < 0.05.
