# Supplementary figures and images for: A novel β2-AR/YB-1/β-catenin axis mediates chronic stress-associated metastasis in hepatocellular carcinoma
Source: Oncogenesis. 2020 Sep 24;9(9):84. doi: 10.1038/s41389-020-00268-w (PMC7515897; doi:10.1038/s41389-020-00268-w)

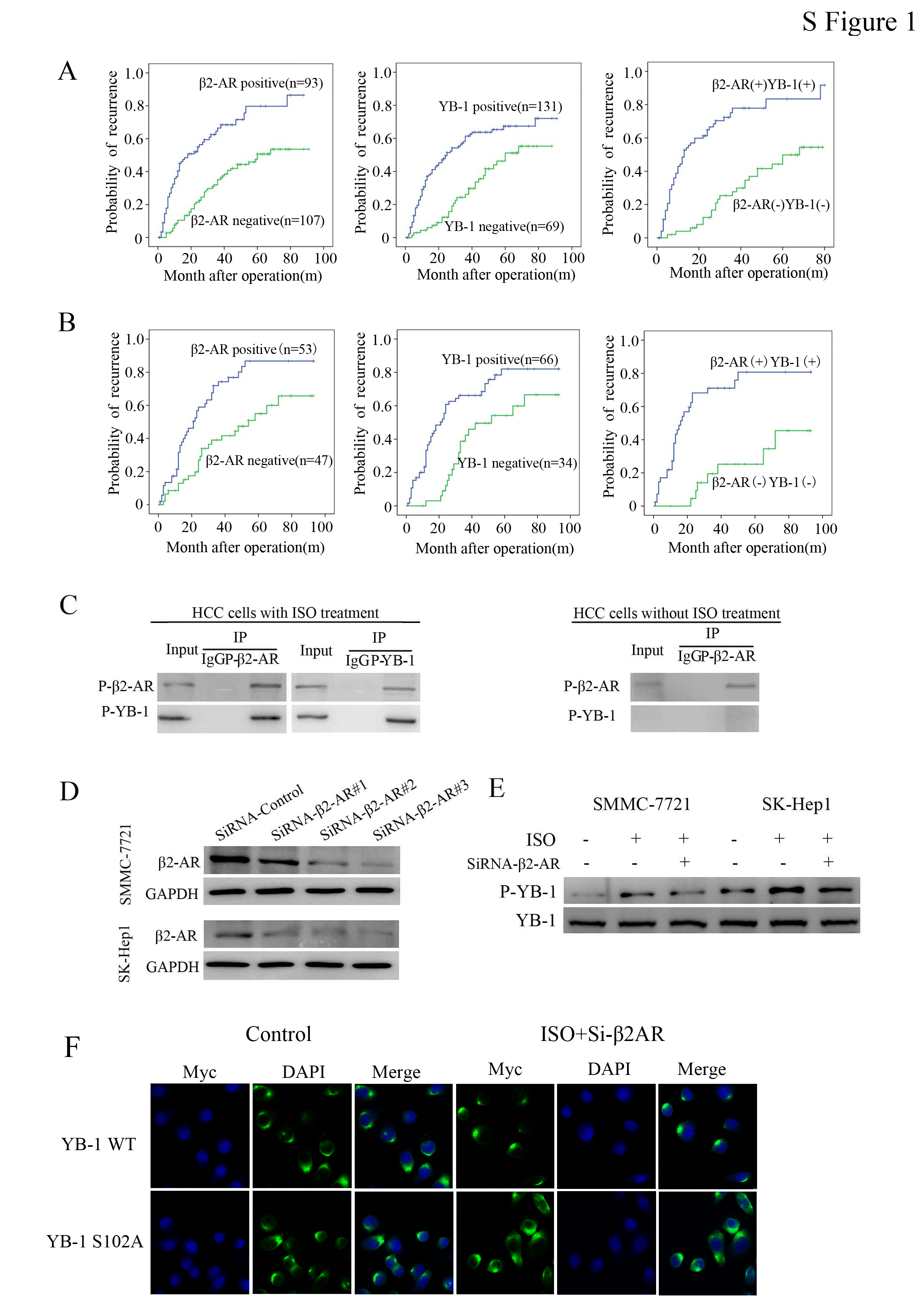

Supplement: Supplementary file 2 — Supplementary Figure 1 [file 41389_2020_268_MOESM2_ESM.tif]

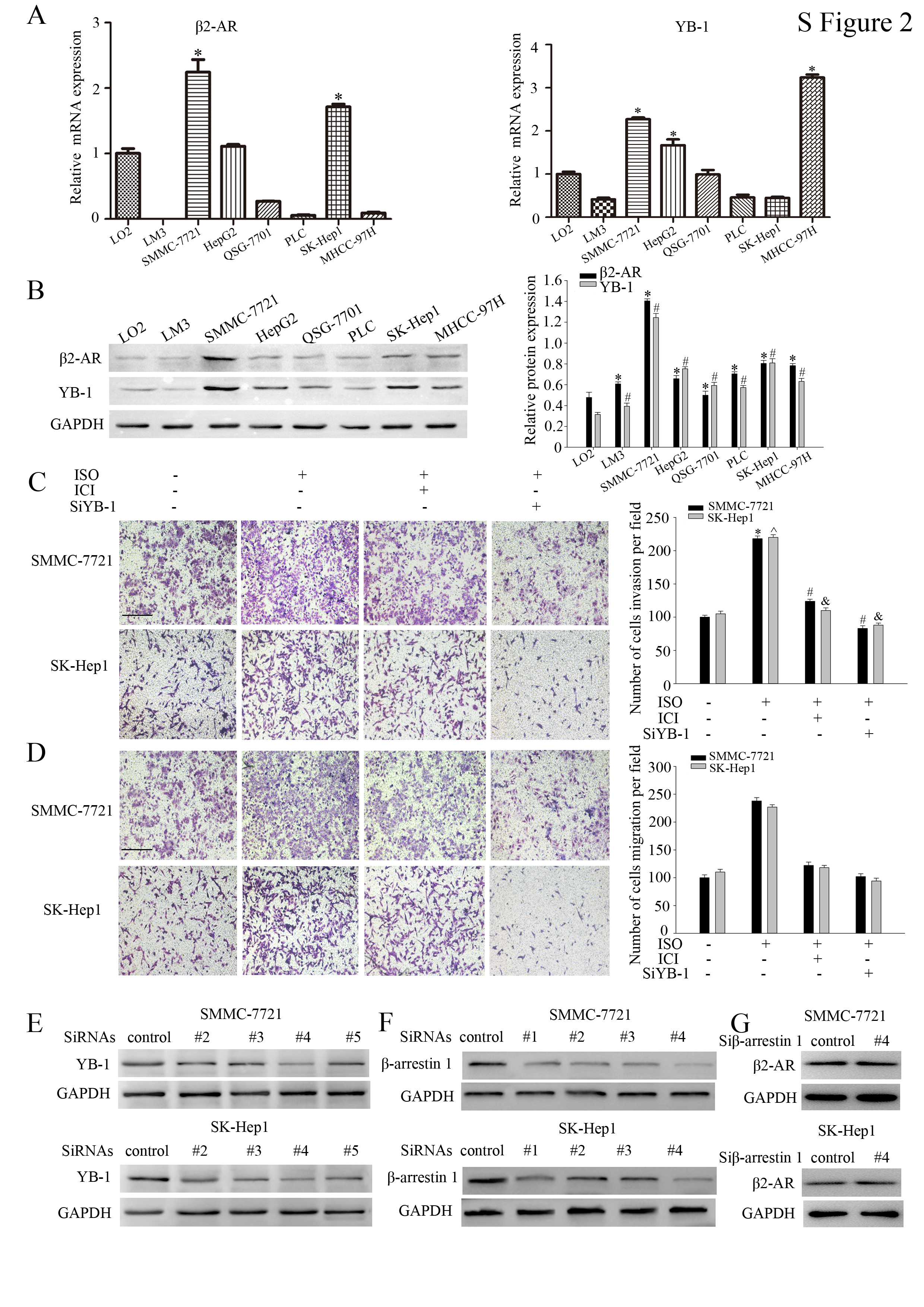

Supplement: Supplementary file 3 — Supplementary Figure 2 [file 41389_2020_268_MOESM3_ESM.tif]
