# Supplementary material for: A novel β2-AR/YB-1/β-catenin axis mediates chronic stress-associated metastasis in hepatocellular carcinoma
Source: Oncogenesis. 2020 Sep 24;9(9):84. doi: 10.1038/s41389-020-00268-w (PMC7515897; doi:10.1038/s41389-020-00268-w)
Supplement: Supplementary file 4 — Supplementary Figure legends [file 41389_2020_268_MOESM4_ESM.docx]

**Supplementary Figure Legends**

**Figure S1.** **a** Kaplan-Meier analysis of the correlation between β2-AR and YB-1 expression and recurrence of HCC patients in cohort I(*P* < 0.05, log-rank test). **b** Kaplan-Meier analysis of the correlation between β2-AR and YB-1 expression and recurrence of HCC patients in cohort II(*P* < 0.05, log-rank test). **c** The interaction between P-β2-AR and P-YB-1 using anti-P-β2-AR and anti-P-YB-1 antibodies, respectively, in SMMC-7721 cells in the presence or absence of 10 μM ISO. **d** Representative Western blot images showing that β2-AR expression in SMMC-7721 and SK-Hep1 cells was significantly decreased by treatment with siRNA-β2-AR #3. **e** Western blotting to detect of total YB-1 and phosphorylated YB-1 (S102) proteins in HCC cells after ISO treatment with or without β2-AR knockdown at 3hours. **f** Confocal immunofluorescence microscopy showed that β2-AR knockdown inhibited ISO-induced nuclear translocation of YB-1 in SMMC-7721 cells. Scale bar: 50 μm.

**Figure S2.** The interaction between β2-AR and YB-1 promotes the invasion and migration of HCC cells. **a** qRT-PCR analysis of β2-AR and YB-1 mRNA levels in HCC cell lines and normal hepatocytes (LO2). The data were mean ± SEM of three independent experiments (**, P <* 0.05) **b** Western blot analysis of β2-AR and YB-1 proteins in different HCC cell lines and normal hepatocytes (LO2). The data were mean ± SEM of three independent experiments (**, P <* 0.05) **c, d** SMMC-7721 and SK-Hep1 cells were pre-incubated with 100 μM ICI118,551 or transfected with YB-1 siRNA and subjected to a cell invasion assay and migration assay in the presence or absence of 10 μM ISO. The data were mean ± SEM of three independent experiments (*, ^#^, ^, &, *P* < 0.05). Scale bar: 100 μm. **e** Representative Western blot images showing that YB-1 expression in SMMC-7721 and SK-Hep1 cells was significantly decreased by treatment with siRNA-YB-1 #4. **f** Representative Western blot images showing that β-arrestin-1 expression in SMMC-7721 and SK-Hep1 cells was significantly decreased by treatment with siRNA-β-arrestin-1 #4. **g** The expression of β2-AR after the downregulation of β-arrestin-1 #4.

**Supplementary Materials and Methods**

**Plasmid construction and cell transfection**

Complementary DNA (cDNA) encoding β2-AR was amplified from a human embryonic brain cDNA library using PCR and then subcloned into pcDNA3.1-HA (+),a mammalian expression vector (Invitrogen), using suitable restrictive enzymes. The pDEST-myc-YBX1 plasmid was a gift from Thomas Tuschl (Howard Hughes Medical Institute, The Rockefeller University, New York, USA) (Addgene plasmid # 19878). The β2-AR and YB-1 deletion mutants were constructed by PCR amplification of the indicated regions and separately cloned into pcDNA3.1-HA (+)/pcDNA3.1-Myc-His (+) vectors. An S102A YB-1 mutant was also constructed. The β-arrestin-1-siRNAs were purchased from GenePharma. The β-arrestin-1 siRNA target sequences were as follows: scrambled, 5′- TTCTCCGAACGTGTCACGT -3′, β-arrestin-1-siRNA1, 5′- CAGTAGATACCAATCTCAT -3′; β-arrestin-1-siRNA2, 5′- TAGCCAATAACCGAGAGAA -3′; β-arrestin-1-siRNA3, 5′- GATCTATTACCATGGAGAA -3′; β-arrestin-1-siRNA4, 5′- CCTTTGAGATCCCTCCAAA -3′. The YB-1-siRNA target sequences were as follows: scrambled, 5′- TTCTCCGAACGTGTCACGT -3′; YB-1-siRNA2, 5′- CGGCAATGAAGAAGATAAA -3′; YB-1-siRNA3, 5′- TAACCATTATAGACGCTAT -3′; YB-1-siRNA4, 5′- AGTTCAAGGCAGTAAATAT -3′; YB-1-siRNA5, 5′- TCAATGTAAGGAACGGATA -3′. Transfection was performed using Lipofectamine™2000 transfection reagent (Invitrogen) in accordance with the manufacturer’s protocol. Forty-eight hours after transfection, the cells were used for subsequent experiments.

**Subcellular fractionation**

Cells were lysed in a lysis solution for 30 min at 4 °C on a rocking platform after two washes in ice-cold PBS. The cells were then broken using a Dounce homogenizer for 10 seconds, and nuclei were pelleted by centrifugation at 3500 × g for 10 min at 4 °C. The cytoplasmic fraction was gathered after centrifugation of the nuclei-free supernatant at 14,000 × g for 10 min at 4 °C to separate the membrane. The nuclear pellets were re-suspended in nuclear lysis buffer (10 mM Tris-HCl, pH 7.5, 150 mM NaCl, 5 mM EDTA, and 1% Triton X-100) for 30 min at 4 °C on a rocking platform. The nuclear fractions were collected by centrifugation of the extraction at 14,000 × g for 10 min at 4 °C.

**Luciferase reporter assay**

SK-Hep1 cells were plated in a 24-well plate for 24 h before transfection. The full-length luciferase reporter with the sequence of the β-catenin promoter or its truncated plasmids (200 ng) along with Myc-tagged YB-1 (200 ng) and 20 ng of pRL-CMV (Promega, Madison, Wl) reference vector were transfected into HCC cells. Forty-eight hours after transfection, HCC cells were washed twice with PBS and subjected to dual-luciferase reporter assay using a Dual-Luciferase Assay kit (Promega) according to the manufacturer’s instructions. The luciferase activities were determined relative to Renilla activity using a ModulusTM TD20/20 Luminometer (Turner Biosystems, USA).

**Transwell assay**

Cell migration and invasion assays were performed in a Transwell chamber (24-well type, 8 mm pore size, Corning, NY, USA). BD Matrigel Basement Membrane Matrix was used according to the manufacturer’s protocol. Cells were then fixed with paraformaldehyde and stained with crystal violet to visualize the nuclei. The results of three independent experiments were averaged.
